# Choledochal cyst- unusual presentation in the adult phase: Case report

**DOI:** 10.1016/j.ijscr.2020.03.014

**Published:** 2020-04-02

**Authors:** Victor Vinicius Monteiro Lins de Albuquerque, Frank Pinheiro De Macedo, Ketlen G. Costa, Zuriel Rodrigues Seixas Nunes, Rubem A. da Silva Junior

**Affiliations:** a Digestive System Surgery Service at Getúlio Vargas Teaching Hospital (HUGV), Avenida Apurinã, 4 - Praça 14 de Janeiro, Manaus, Amazonas, 69020-170, Brazil; b General Surgery Service at Getúlio Vargas Teaching Hospital (HUGV), Avenida Apurinã, 4 - Praça 14 de Janeiro, Manaus, Amazonas, 69020-170, Brazil; c Medical School of Federal University of Amazonas (UFAM), Rua Afonso Pena, 1053 - Praça 14 de Janeiro, Manaus, Amazonas, 69020-160, Brazil

**Keywords:** Choledochal cyst, Todani’s classification, Hepaticojejunal anastomosis

## Abstract

**Introduction:**

Bile duct cysts are congenital malformations characterized by bile duct dilatation with intra and/or extrahepatic localization. About 80% of cases are diagnosed in childhood, so their presentation in adults is rare and repeatedly associated with complications.

**Presentation of case:**

A 38-year-old female patient complained of severe abdominal pain for one month, with no associated cholestatic signs. She underwent abdominal ultrasonography that showed cholelithiasis with extrahepatic bile duct dilatation and underwent cholangiography (MRI), which revealed fusiform dilatation of the proximal portion of the hepatocholedochal, compatible with choledochal cyst.

**Discussion:**

In adult patients, clinical manifestations are usually nonspecific, and the diagnosis is established by medical imaging. Magnetic resonance cholangiopancreatography (MRCP) is the gold standard diagnostic today. Treatment with better prognosis consists of total cyst resection with hepaticojejunal anastomosis and intestinal transit reconstruction with Roux-en-Y.

**Conclusion:**

Bile duct cysts are a rare condition in adults. Currently, increasing access to more accurate medical imaging enables early diagnosis, contributing to the use of therapeutic methods in appropriate time, avoiding development of complications.

## Highlights

• Bile duct cysts are a rare condition in adults.
• The classic triad of signs and symptoms is rarely present.
• The main treatment is surgical resection of the cyst(s).

## 1 Introduction

Bile duct cysts are congenital malformations characterized by biliary duct dilatation with an intra and/or extrahepatic localization. There are incidences of 1:100.000 to 150.000 live births in the West and 1:1.000 in Asia, being most prevailing in female at a ration from 3 to 4:1 [[1], [2], [3], [4], [5], [6], [7], [8]]. Around 80% of cases are diagnosed in childhood, thence, the presentation in adults is rare and it is repeatedly associated with complications such as cholangitis, stone formation, cyst rupture, secondary biliary cirrhosis, obstructive jaundice and malignancy (cholangiocarcinoma) [1,3,9,10]. The typical clinical presentation features abdominal pain, jaundice and palpable mass in the right upper quadrant. However, in adults the presentation can be difficult with nonspecific abdominal pain, under consideration the classic triad observed in about 25% of cases for this group [1,3,10,11]. With the development and improvement of medical imaging, it was made possible the diagnosis of a growing number cases. Amongst the main requested investigations, abdomen ultrasonography, computed tomography (CT), magnetic resonance cholangiopancreatography (MRCP), endoscopic retrograde cholangiopancreatography (ERCP) and the endoscopic ultrasound are also present [1,4,5,12,13]. The treatment with a better prognosis consists of a total cyst resection, hepaticojejunal anastomosis and intestinal transit reconstruction with Roux-en-Y technique [1,[4], [5], [6],^10^,[13], [14], [15]]. This work was reported in line with the SCARE criteria [16].

## 2 Report

A 38-year-old female patient from Parintins, Manaus, Amazonas presented with the chief complaint of “belly pain”. She reported that it began a month ago with mild to severe epigastric pain of the colic type, which radiated to the right hypochondrium and back, beginning after feeding, which persisted even after home analgesia. She denies fever or chills, choluria, jaundice, fecal alcoholism, or recent weight loss. Physical examination was without semiological changes. She underwent abdominal US that revealed cholelithiasis with extrahepatic bile duct dilatation and thus requested MRI and referred to the general surgery outpatient clinic. She denied any other complaints, such as comorbidities, medication use, allergies or previous surgery.

**Fig. 1:**
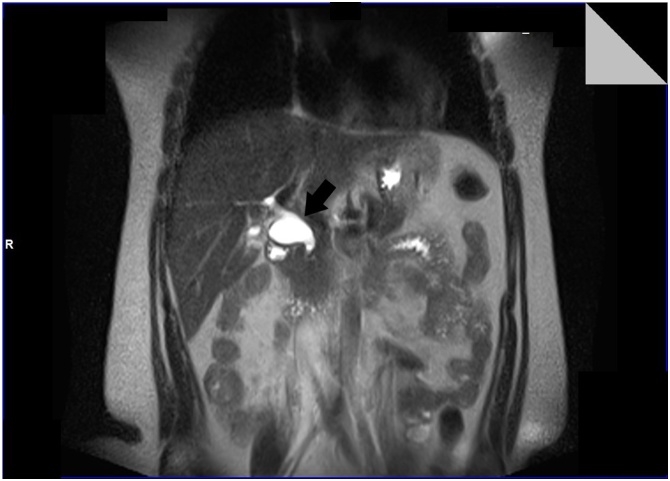
MRI showing fusiform dilatation of the proximal portion of the hepatocholococcus (arrow), measuring up to 2.5 cm, compatible with Todani type I choledochal cyst.

Imaging (MRI) revealed normal-walled gallbladder with multiple thin septations within it, notably in the body and fundus, as well as fusiform dilation of the proximal portion of the hepatocholedocal duct, measuring up to 2.5 cm, compatible with cyst of Todani type I choledococcus (Fig. 1).

**Fig. 2:**
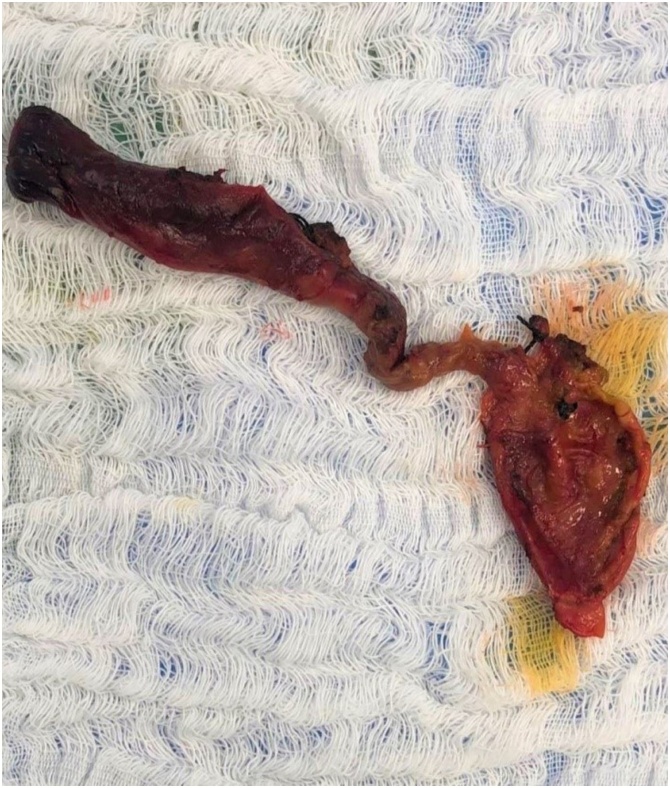
Surgical specimen after resection of the main biliary tract.

After two months, the patient underwent cholecystectomy and complete surgical resection of the extrahepatic bile duct cyst segment to about 1 cm below the bifurcation of the hepatic ducts, followed by Roux-en-Y end-lateral hepatic jejunostomy (Fig. 2). The postoperative evolution was satisfactory, being discharged on the eighth day after surgery, being followed so far without evidence of complications.

## 3 Discussion

Reports of bile duct cysts have been gone up in recent years, with more than half of the cases occurring in Japan. A report from Finland estimates that the incidence of bile duct cysts has been increased from 1:128.000 to 1:38.000 for the past 40 years; and although most cases were reported in children in the past, some studies have registered similar data in adults and children [11]. Despite various theories created, the exact aetiology remains uncertain. The most accepted one is the proposal by Babitts in 1969 [17], in which states that choledochal cysts are caused by an anomalous junction of the pancreatic and choledochal duct outside the duodenal wall, proximal to Oddi’s sphincter during the embryonic period; with a formation of a common duct about 15 mm and that generates reflux from pancreatic bile duct secretions [1,[3], [4], [5], [6], [7], [8], [9],[11], [12], [13],^17^,^18^]. The activation of pancreatic enzymes in the biliary drainage system generates an increased intra-ductal pressure and inflammation, leading to a dilatation with cyst formation [7,8,18]. Despite being common, representing 57–96% of cases, it does not represent the etiologic factor from all cases of biliary cysts [5].

Regarding classification, Alonso-Lej and colleagues elaborated the first system in 1959, which encompassed 4 types of biliary cysts (type I to IV) [19]. In 1977, Todani and colleagues made modifications and added a fifth category characterized by the presence of intrahepatic cysts (Type V or Caroli Disease) [20]. In 2003 the presence of anomalous pancreaticobiliary junction was added to a classification modified by Todani, in which is currently the most utilized one [11]. It was summarized in Table 1 and it divides the patients in 5 groups according to place, extension and shape of cystic alterations [1,[3], [4], [5], [6], [7], [8], [9],[11], [12], [13],^19^].

**Table 1:** Todani modified Alonso-Lej classification for bile duct cysts with the respective frequencies.

Type I (50–85%)	Cystic or fusiform dilatation of the bile duct without affecting intrahepatic bile duct.
	**Type IA:** Cystic dilatation of the common bile duct, as well as part of the hepatic common duct and some portions from the right and left hepatic duct.
	**Type IB:** segmental/focal dilatation of the extrahepatic common bile duct.
	**Type IC:** fusiform dilatation of the extrahepatic biliary tree.
	**Type ID:** cystic dilatation of the common and cystic duct.
Type II (2%)	Real diverticulum of the extrahepatic bile duct.
Type III (1 a 5%)	Choledococeles
	**Type IIIA:** the common bile duct and the pancreatic duct enter in the cyst which connects to duodenum through another orifice.
	**Type IIIB:** an intra-ampullary common bile duct diverticulum
Type IV (15–35%)	Presence of multiple intrahepatic and extrahepatic cysts, or only the extrahepatic ones.
	**Type IVA:** intra and extrahepatic dilatations.
	**Type IVB:** multiple dilatations in the extrahepatic bile ducts only.
Type V (20%)	One or more cystic dilatations of the intrahepatic biliary tract (without involvement of extrahepatic biliary duct).
Type VI (rare)	Isolated dilatation of the cystic duct.

Concerning the clinical presentation, there is no consensus in the literature about the most common one. Some authors affirm that jaundice is the most observed symptom [2], whereas others claim to be an abdominal pain [12]. Both symptoms added up to the presence of palpable abdominal mass in the right upper quadrant make up the classic triad seen in a minority of patients (about less than 20%), with reports of higher expression in the pediatric age group [3,5,6,[8], [9], [10]]. In adult patients, the initial manifestations are unspecific, ranging from right upper quadrant abdominal pain, fever, nausea, vomiting to jaundice [1,11,13]. In children, about 85% have at least 2 classic triad symptoms [1].

The diagnosis of bile duct cysts is defined by the identification of bile duct dilation after exclusion from other etiologies (neoplastic, calculus or inflammatory) [3]. This condition can be identified by using specific imaging exams, such as ultrasound (US), computed tomography (CT), magnetic resonance cholangiopancreatography (MRCP), endoscopic retrograde cholangiopancreatography (ERCP) and endoscopic ultrasound [[1], [2], [3], [4], [5], [6],^8^,^9^,[11], [12], [13]]. In most cases ultrasound is the first examination performed to investigate the condition, being effective to evaluate the presence of dilatations and gallstones in the biliary duct [3,10]. In case of suspicion for the diagnosis of biliary cyst at a US, a CT or MRCP is requested; the last one, however, would be considered gold standard these days and the main choice for diagnostic evaluation, as it is a non-invasive technique which does not use radiation ionizing agent [2,5,6,8,9,11].

The standard treatment from bile duct cysts is surgical with specific approaches for each type according to its classification. Those classified as type I, II or IV are generally undergone resection surgery due to the risk of neoplastic development. The types I and V are completely resected with cholecystectomy completion and biliary reconstruction by Roux-en-Y hepaticojejunostomy, being required a partial hepatectomy on IVA type [3,11]. The technique previously mentioned is considered a gold standard for traffic reconstruction in pathologies which affect the bile ducts, with studies demonstrating dehiscence rates between 2% to 6%. Colodecoduodenostomy or hepaticoduodenostomy configure other possible approaches, however, studies have shown that the occurrence of biliary gastritis was a recurring consequence to this technique when used for benign conditions treatment [21]. Types II are treated with cystic lesion resection and in specific cases, it may be required extension resection associated with biliary [4,9,11] transit reconstruction. In type IIIA cysts, it is recommended endoscopic sphincterectomy whilst types IIIB can be resected surgically or endoscopically [1,4,9,11]. Those classified as type V require a multidisciplinary approach (endoscopy, interventional radiology and surgery). In some cases, the only possible treatment is supporting, in order to prevent development from cholangitis and sepsis. Nevertheless, in other cases it is possible to use surgical approaches such as: segmental liver resection, lobar resection or liver transplantation [1,3,4,9,11].

## 4 Conclusions

Bile duct cysts are rare pathology in adults, happening to be more common in the pediatric age group, especially in Asian population. Currently, improvements in health care and the increase in accessing more accurate medical imaging enable early diagnosis, contributing to use of therapeutic methods in appropriate time, avoiding development complication. The classic triad signs and symptoms are rarely present, as such vital the awareness of the disease to include it in diagnostic hypothesis and make use of optimal screening tests with a favourable cost-benefit, hence ultrasound to be the most used in the initial approach. The main treatment is consisted in surgical resection from cyst(s) when feasible, due to the risk of future malignancy.

## Funding

We do not have any funding source, this manuscript is just a case report, not a research.

## Ethical approval

As the manuscript is not a research study, we only have the patient consent for writing and others forms of publication. Also, the ethical approval for this case report has been exempted by our institution.

## Consent

Written informed consent was obtained from the patient for publication of this case report and accompanying images. A copy of the written consent is available for review by the Editor-in-Chief of this journal on request.

## Author contribution

Zuriel Seixas and Ketlen Sousa made contributions to conception and design. collected the patient details and wrote the paper. Frank Macedo made contributions to patient management. Victor Albuquerque and Rubem Silva Junior critically revised the article. All authors read and approved the final manuscript.

## Registration of research studies

The manuscript is a case report, not considered a formal research involving participants.

## Guarantor

Dr. Victor Albuquerque.

## Acknowledgement

None.

## Provenance and peer review

Editorially reviewed, not externally peer-reviewed.

## Declaration of Competing Interest

The authors declare that there are no conflicts of interest.
